# Research on Sol-Gel Synthesis of Low-Temperature Na_2_O-B_2_O_3_-SiO_2_ Vitrified Bonds and Preparation of High-Strength Stacked Abrasives Using the Molding and Crushing Method

**DOI:** 10.3390/ma17081799

**Published:** 2024-04-14

**Authors:** Pei Wang, Lingrui Liang, Zhihong Li, Yumei Zhu

**Affiliations:** Key Laboratory for Advanced Ceramics and Machining Technology of Ministry of Education, School of Materials Science and Engineering, Tianjin University, Tianjin 300072, China; 2021208150@tju.edu.cn (P.W.); lianglingrui@tju.edu.cn (L.L.); lizhihong@tju.edu.cn (Z.L.)

**Keywords:** sol-gel, stacked abrasives, compressive strength, low-temperature vitrified bonds

## Abstract

Currently, the sol-gel technique is employed in the synthesis of high-performance vitrified bonds; however, its application in the fabrication of stacked abrasives has been minimally explored. Furthermore, the methods utilized in the production of abrasive particles for stacked abrasives are technically challenging and incur high costs, which hinders their actual industrial application. Consequently, this study utilizes the sol-gel approach to synthesize a Na_2_O-B_2_O_3_-SiO_2_ ternary system vitrified bond powder and employs a molding and crushing method, which offers a lower technological barrier and reduced preparation costs, for the production of abrasive particles subsequently fabricating corundum stacked abrasives. Upon setting the binder composition to a molar ratio of n(SiO_2_):n(B_2_O_3_):n(Na_2_O) = 65:23:12, it was observed that the crystallization within the glass matrix was minimized and the optimal sintering temperature for the synthesized laminate abrasive to be sustained at 820 °C. At the aforementioned temperature, the binder melt is capable of flowing uniformly amongst the abrasive granules, thereby ensuring a robust encapsulation of the particles. The average single particle compressive strength of the prepared corundum stacked abrasive with a grain size of forty mesh can reach the highest of all composition points at 28.56 N and the average single particle compressive strength of the prepared diamond stacked abrasive is 28.14 N.

## 1. Introduction

Against the backdrop of rapid advancements in cutting-edge technologies, such as aerospace, high-efficiency and high-precision machining and grinding technology has become the current research focus [1,2,3]. Traditional single-grain abrasives perform poorly in terms of grinding efficiency and workpiece surface consistency, and they are no longer able to meet the development requirements of high-precision, high-speed, and high-efficiency grinding technology [4,5].

As a new type of abrasive material in the grinding industry, stacked abrasives are a type of composite material formed by combining fine abrasive powders with high-performance bond powder and through specific processes, such as mechanical mixing or spray drying granulation, and then sintering at a certain temperature, to form specific shapes. Compared to traditional abrasives, stacked abrasives have higher inter-particle bonding strength, controllable shapes, and can achieve micro-cutting and multipoint surface contact grinding during the machining process [6,7]. Therefore, while significantly improving the service life of grinding tools, they also greatly enhance the precision and surface quality of grinding processes [8], thus finding wide applications in precision machining and becoming a research hotspot in the abrasive field [9].

Grinding tools consist of abrasives, bonds, and pores, among which the vitrified bond, which acts to bond abrasive grains, has an extremely important influence on the performance of the grinding tool [10,11,12]. Vitrified bonds can be divided into two types: traditional mineral bonds and vitreous bonds. Traditional mineral bonds use complex mineral raw materials, have high firing temperatures, and the use of mechanical mixing methods can lead to uneven melting of the glass phase due to different softening temperatures and firing temperatures of each component during the calcination process, affecting the performance of the bond. The main difference between stacked abrasives as a type of micro-tool and conventional grinding tools lies in the much smaller particle size of the abrasive micro-powders used in stacked abrasives, ranging from 0 to 10 μm in diameter. To bond these fine micro-powders, coupled with the tendency of diamond to graphitize at high temperatures, vitrified bonds must not only have good low-temperature sintering performance and a small particle size, but also excellent phase uniformity [13,14] to ensure the performance of stacked abrasives. This is far from sufficient for traditional mineral bonds, which must be at high temperatures to exhibit good sintering properties and have uneven phase compositions [15].

Therefore, people later used the melt quenching method to directly prepare vitrified bond powders with the glass phase. Since the raw materials initially melted at high temperatures to form a liquid phase for thorough mixing and reaction, the phase homogeneity of the vitrified bond powder increased to some extent. Additionally, since the vitrified bond prepared in this way is amorphous glassy phase, its softening temperature is low, thus effectively reducing the sintering temperature. However, glassy vitrified bonds prepared by melt quenching generally require thorough melting of the raw materials at temperatures above 1200 °C to ensure uniform mixing, which consumes a considerable amount of energy [16,17]. Therefore, it is extremely important to develop new vitrified bonds that can be sintered at low temperatures, have superior performance, and are more energy-efficient and environmentally friendly.

The sol-gel method controls material properties by adjusting parameters, such as sol composition, concentration, temperature, and pH value. Inorganic materials prepared by this method have advantages such as high purity, uniformity, controllable morphology, and diverse structures, and have been widely used in catalysts, electrochemical materials, sensors, separation membranes, microcrystalline corundum abrasives and other fields [18,19,20,21,22]. Additionally, the sol-gel method plays an important role in the preparation of inorganic glasses, providing an effective preparation method for the research and application of inorganic glasses [23,24]. According to previous studies, in addition to energy-saving and environmentally friendly processes, glassy powders prepared by the sol-gel method have advantages such as small particle size, chemical uniformity, and high purity [25,26]. Some studies have used this method to prepare glassy vitrified bonds for the production of stacked abrasives. The results show that the sol-gel bonds have higher sintering activity, allowing for the production of high-quality stacked abrasives with low sintering temperatures, high compressive strength, and uniform internal structure. However, most of these studies currently use spray drying [27] or inverse microemulsion polymerization [28], which have high technical difficulty and preparation costs, indicating that true industrial production is still some distance away. Therefore, this study employs the sol-gel method to prepare vitrified bond powders of Na_2_O-B_2_O_3_-SiO_2_ with lower sintering temperatures and basic characteristics and adopts the molding and crushing method with low technical barriers and preparation costs to prepare corundum stacked abrasives and diamond stacked abrasives. The study analyzed the thermal analysis curve and crystallization changes of the dried gel, and systematically investigated the effects of compositional changes on the crystallization of binders and the optimal sintering temperature, and the effects of sintering temperature and bond addition on the single-particle compressive strength and microstructure of corundum-stacked abrasives. Ultimately, the ingredient ratio with fewer crystallizations, lower sintering temperatures, and higher strengths were obtained, resulting in diamond-stacked abrasives with high single particle compressive strength.

## 2. Materials and Methods

### 2.1. Sample Preparation

#### 2.1.1. Selection of Component Points

According to the theory of glassy amorphous network and current research on borosilicate glass [29,30,31]: both silicon dioxide and boron oxide can individually form glass, but due to the tetrahedral structure formed by [SiO_4_], and the layered structure formed by [BO_3_], it is difficult to form a uniformly consistent melt between these two oxides. The addition of Na_2_O provides free oxygen to the glass system. Free oxygen causes [BO_3_] to transform into [BO_4_], thereby transforming the layered structure of boron oxide into a framework structure, thereby enhancing the strength of the glass. Therefore, under constant preparation conditions, the relative content of Na_2_O and B_2_O_3_ in borosilicate glass will significantly affect the quantity and relative transformation of [BO_3_] and [BO_4_] in the glass system. Generally, in Na_2_O-B_2_O_3_-SiO_2_ glass systems, an increase in the quantity of free oxygen promotes the transformation of [BO_3_] to [BO_4_] in the glass system. Based on the Na_2_O-B_2_O_3_-SiO_2_ ternary phase diagram glass formation area, the melting coefficient empirical formula and experimental attempts, seven component points, whose sintering temperature can be maintained in the range of 760 °C to 880 °C, were selected and numbered as shown in Table 1.

#### 2.1.2. Preparation of Vitrified Bond Powder by Sol-Gel Method

The vitrified bond powder was prepared using the sol-gel method. Raw materials such as ethyl silicate (TEOS), boric acid (H_3_BO_3_), and sodium nitrate (NaNO_3_) were used, along with solvents like ethanol (EtOH) and deionized water. Nitric acid was used as a catalyst. The molar ratio of n (H_2_O)/n(TEOS) = 50:1, and the molar ratio of n (EtOH)/n (TEOS) = 8:1. Boric acid and sodium nitrate aqueous solutions were added dropwise into the ethanol solution of ethyl silicate, followed by the addition of the catalyst to adjust the pH to 3.5. The mixture was stirred for 2 h to obtain a sol. The sol was dried at 100 °C for 36 h to obtain a dry gel, which was then subjected to heat treatment at 600 °C followed by ball milling and sieving through a 200-mesh sieve to obtain the final sol-gel vitrified bond powder.

#### 2.1.3. Preparation of Vitrified Bond Powders by Dry Mixing Method

Analytical grade sodium carbonate, silicon dioxide, and boric acid were used as raw materials. They were weighed according to the formulation ratio, poured into a ball mill, and milled at a speed of 300 r/min for 2 h to achieve uniform mixing. Subsequently, the mixture was sieved through a 300-mesh sieve to obtain vitrified bond powder prepared by the dry mixing method.

#### 2.1.4. Preparation of Stacked Abrasives by Molding and Crushing Method

**Figure 1:**
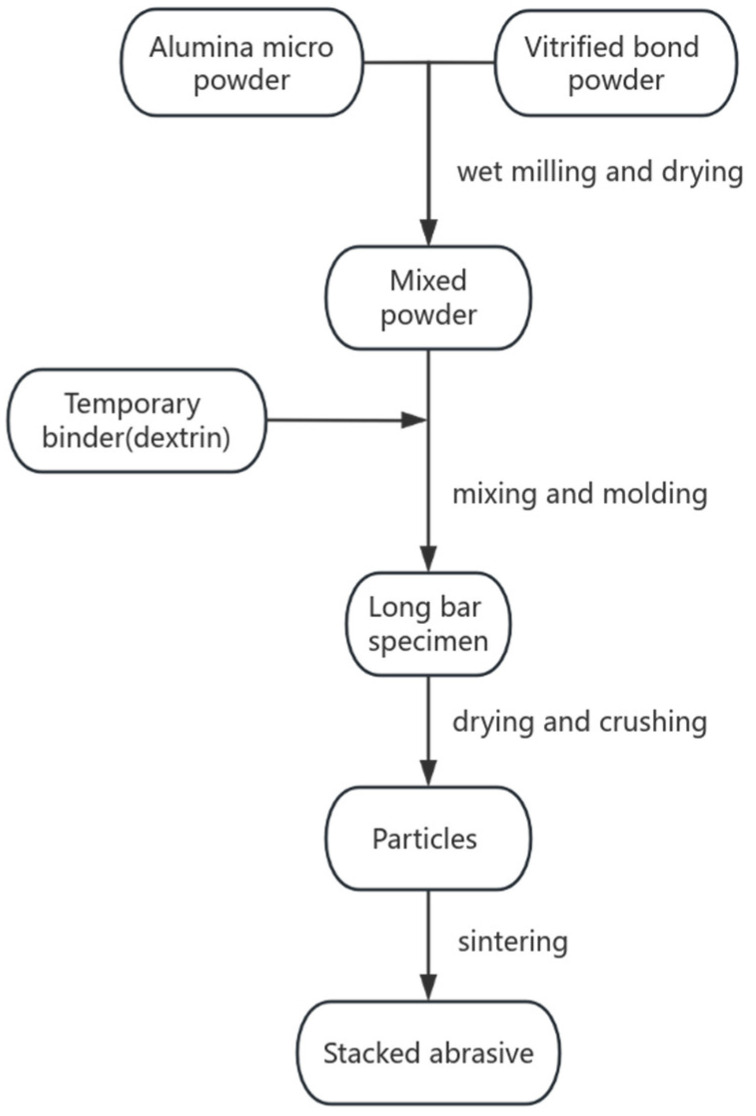
Preparation of stacked abrasives by molding–crushing method.

The specific process is shown in Figure 1. The sol-gel vitrified bond powder is mixed with alumina micro-powder, with a particle size of W1 according to the mixing ratio, and then placed in a ball mill for wet grinding at a speed of 300 r/min for 2 h to achieve uniform mixing. Subsequently, after drying to remove moisture, the powder suitable for subsequent forming is obtained. Temporary binder (gelatin solution) is added to the powder at a ratio of 5% of the mass of the powder and mixed uniformly. Then, under the condition of molding density of 2.37 g/cm^3^, the material is pressed into rectangular bars with dimensions of 30 × 6 × 6 mm using a press. Subsequently, the bars are placed in a drying oven and dried at 70 °C for 2 h to give them a certain strength for subsequent crushing. Then, the bars are crushed into particles of specified particle size using a mortar and pestle, and these particles are placed in a muffle furnace and sintered at a certain temperature for 2 h to obtain corundum stacked abrasives. Subsequent diamond stacked abrasives are prepared using this process, as well.

### 2.2. Sample Preparation

The thermal behavior of the dried gel powder was analyzed using a thermal analyzer (STA 449F3, NETZSCH, Selb, Germany) in the temperature range from 20 °C to 1000 °C under an air atmosphere with a heating rate of 10 °C/min.

The phase composition of the vitrified bond powder under different temperature treatments was analyzed using an X-ray diffractometer (XRD, D8 Advanced, Bruker, Ettlingen, Germany), with Cu Kα radiation, scanning range from 10° to 90°, and scanning speed of 10°/min.

Elemental analysis of the microscopic surface of the stacked abrasives was conducted using X-ray energy dispersive spectroscopy (EDS, X-MAX20, OXFORD, Oxford, UK).

Through the scanning electron microscopy (SEM, Hitachi S-4800, Tokyo, Japan), the microstructure of the stacked abrasives and the dispersion of vitrified bonds within the abrasive grains were observed and analyzed.

The single particle compressive strength of the abrasives was measured using a diamond static pressure tester (ZMC-II, Zhengzhou Research Institute of Abrasives & Grinding, Zhengzhou, China). In the experiment, abrasive samples with a particle size of 40 mesh (0.425 mm) were selected, and their average value was calculated. The compressive strength of 40 particles was then measured. Values above twice the average and below half the average were discarded, and the average of the remaining compressive strength measurements was taken as the final result.

## 3. Results and Discussion

### 3.1. Thermal Analysis and Phase Analysis of Bond Powder Prepared by Sol-Gel Method

**Figure 2:**
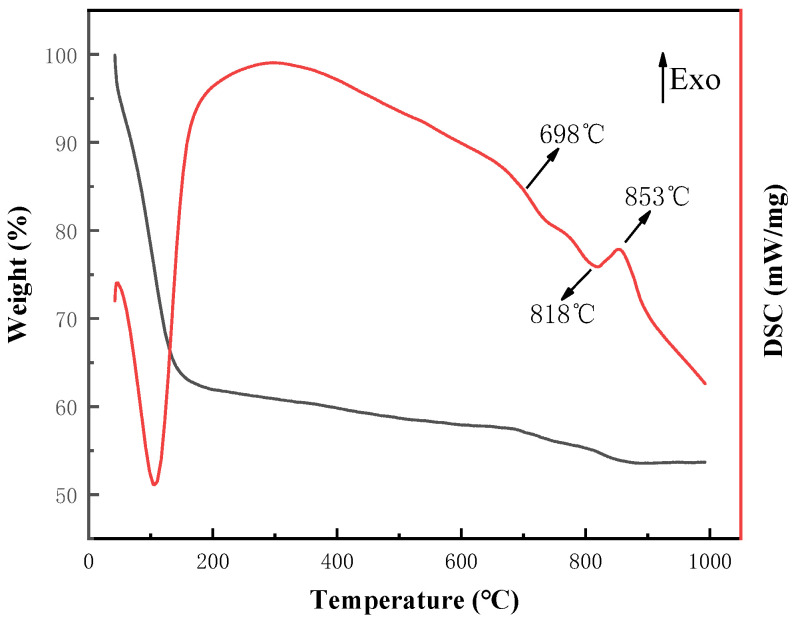
TG-DSC curve of dry gel powder.

**Figure 3:**
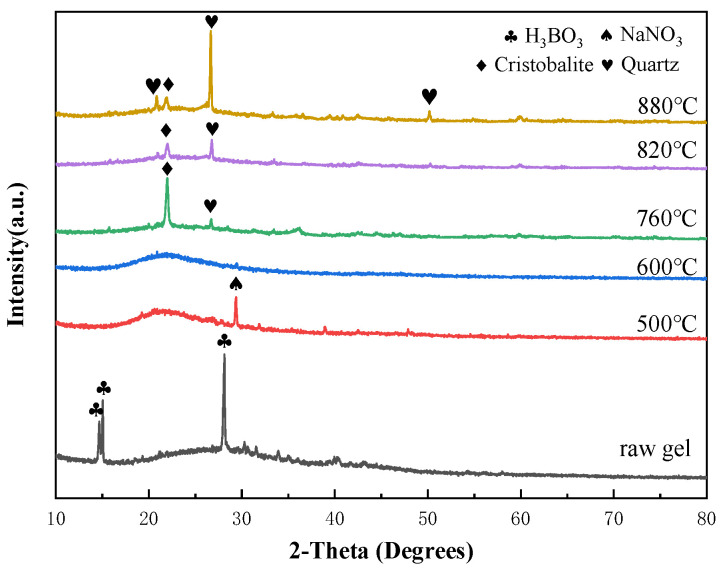
XRD patterns of vitrified bonds sintered at different temperatures.

Thermal analysis and phase analysis at different temperatures were conducted on the sol-gel binder powder of component A2, and the specific results are shown in Figure 2 and Figure 3. A distinct endothermic peak was observed in the temperature range of 40 °C to 200 °C, attributed to the evaporation of surface-adsorbed water and organic solvents. In the range of 200 °C to 600 °C, a broad and intense exothermic peak was observed. This phenomenon is not only caused by the continued evaporation of alcohols and water in the gel system, but it also involves dehydration between organic groups (such as [≡Si-OH] and [=B-OH]), and the detachment of [-OH] groups from [≡Si-OH] and [=B-OH]. It is accompanied by the comprehensive exothermic reaction of borate decomposition and integration into the glassy amorphous structure (in the XRD spectrum, the diffraction peak of boric acid disappears after heat treatment at 500 °C). Additionally, between 500 °C and 600 °C, there is also the decomposition reaction of nitrate, releasing heat (the diffraction peak of sodium nitrate appears at 500 °C, while it disappears at 600 °C, indicating that sodium nitrate has not completely decomposed before 500 °C). Based on the thermal analysis spectrum, the glass transition temperature is approximately 698 °C. Subsequently, according to the XRD spectrum, diffraction peaks of quartz appeared at 760 °C, indicating the crystallization of quartz near this temperature. Between 790 °C and 880 °C, the diffraction peak of quartz gradually decreased, while the diffraction peak of cristobalite increased, indicating a crystallization or phase change within this temperature range, corresponding to the exothermic peak near 853 °C in the thermal analysis curve (730 °C to 890 °C).

**Figure 4:**
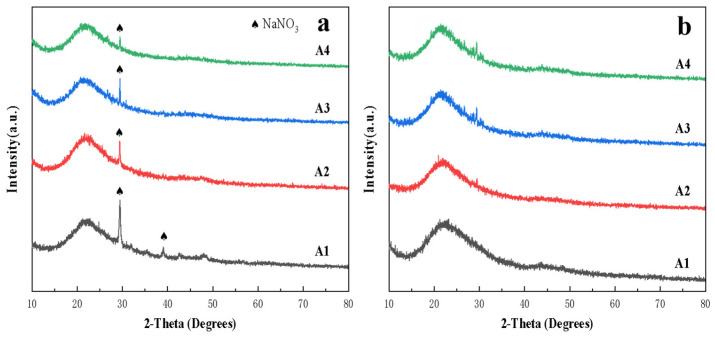
XRD patterns of dry gels at A1 to A4 after heat treatment at 500 °C (**a**) and 600 °C (**b**).

The purpose of the thermal treatment is to remove excess visible physical forms of organic solvents and water, as well as a large amount of nitrate ions, hydroxyl groups, alkyl groups, and other organic substances in the dry gel system. If these organic substances are not thoroughly removed during the gel thermal treatment process, it will adversely affect the performance of the obtained vitrified bond powder. This will lead to the generation of excess pores during the sintering process of the micro-powder and the vitrified bond, which affects the compressive strength of the stacked abrasive. From the previous analysis, it is known that there may still be residual sodium nitrate crystals in the ceramic bond prepared by the sol-gel method after heat treatment at 500 °C. Therefore, in order to determine the heat treatment temperature, X-ray diffraction analysis was carried out after heat treatment at 500 °C and 600 °C for the dry gels prepared at A1 to A4 with different sodium oxide ratios. As shown in Figure 4, the diffraction peak of sodium nitrate is gradually enhanced as the proportion of sodium oxide is increased from A4 to A1. This indicates that the thermal treatment temperature of 500 °C is insufficient to remove the sodium nitrate component from the bond. After thermal treatment at 600 °C, it was found that the diffraction peaks of sodium nitrate in the dry gels of the four component points had basically disappeared, leaving only the amorphous diffraction peaks, confirming the amorphous structure of the sol-gel vitrified bond after thermal treatment. Therefore, 600 °C was chosen as the subsequent thermal treatment temperature for the dry gel.

### 3.2. Single Particle Compressive Strength of Corundum Stacked Abrasives

#### 3.2.1. Effect of Oxide Component Ratios of Vitrified Bonds on the Single Particle Compressive Strength of Corundum Stacked Abrasives

The compressive strength of stacked abrasive particles is influenced by the composition of the bond itself, the sintering temperature and the crystallization behavior. The proportion and content of oxides in the bond composition lead to variations in the glass network structure, which in turn cause differences in the strength of the glass bond; the sintering temperature affects the flow ability of the binder, which influences the degree of movement and encapsulation of the fine powder by the bond. Furthermore, the composition of the bond and the sintering temperature also jointly affect the crystallization of the bond. Crystallization within the glass amorphous structure can lead to weakened grain boundaries and chemical inhomogeneity, resulting in localized stress concentration, which affects the overall strength of the vitrified bond. At a compositional point, where no crystallization or only weak crystallization occurs, the optimal sintering temperature for the best single particle compressive strength is the temperature at which the bond powder achieves its optimal flowability, thus allowing for complete encapsulation of the abrasive particles.

**Figure 5:**
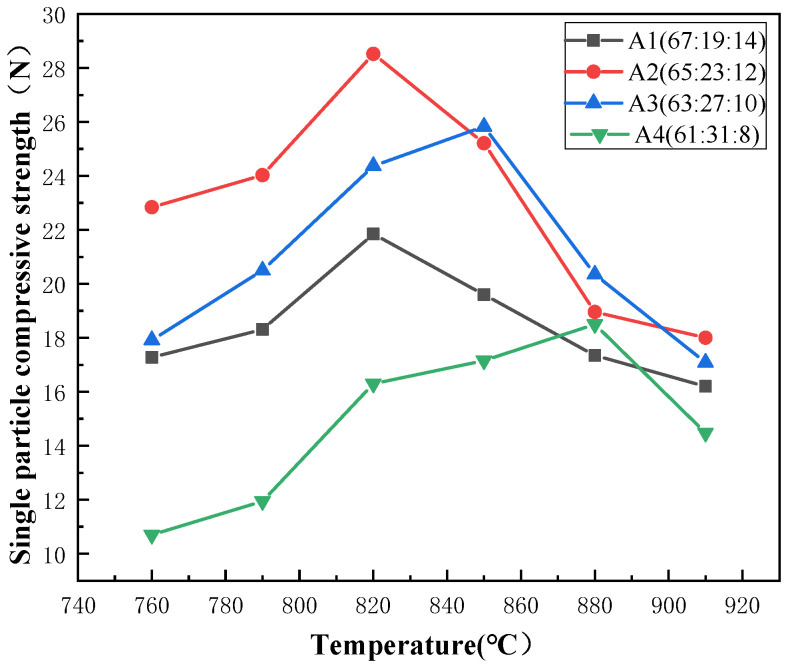
Single particle compressive strength of stacked abrasives prepared at A1 to A4.

**Figure 6:**
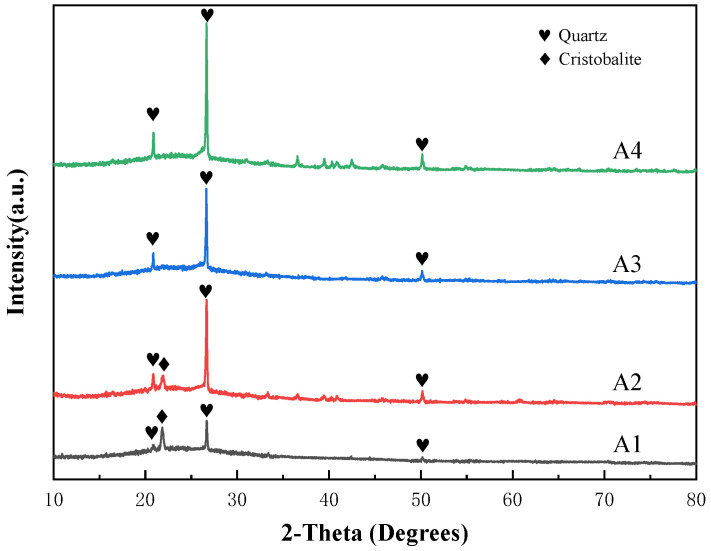
XRD patterns of stacked abrasives prepared at A1 to A4 sintered at 850 °C.

Analysis from Figure 5 below shows that for the four compositional points from A1 to A4, the content of silicon dioxide and sodium oxide decreases sequentially, while the content of boron oxide increases, resulting in a gradual rise in the optimal sintering temperature and an increase in the optimal single particle compressive strength, peaking at 28.56 N for A2, followed by a gradual decrease, with a significant drop to 18.5 N for A4. From Figure 6, the diffraction peaks of the quartz crystals gradually intensify with the change of composition points from A1 to A4, indicating that the amount of crystal precipitation gradually intensifies.

A1 to A2: The optimal sintering temperature for these two composition points remains unchanged at 820 °C. However, the compressive strength of A2 is significantly higher than A1 at each temperature, and as shown in Figure 6, the crystallization amount of A1 is slightly smaller than A2, with little difference between the two composition points. This directly indicates that the higher compressive strength of the stacked abrasives at A2 is due to the improved strength of the glassy network structure compared to A1. Applying current theoretical analysis of borosilicate glass, at composition point A1, B_2_O_3_ exists simultaneously in the glass structure in both [BO_3_] and [BO_4_] forms. The free oxygen provided by the Na_2_O partly serves to cut off the [SiO_4_] network and partly serves to allow the [BO_3_] to exist in [BO_4_] form, so that the glass as a whole is in a state of free oxygen excess. Compared to A1, A2 exhibits an increase in B_2_O_3_ and a decrease in SiO_2_ and Na_2_O. Therefore, in this situation, the newly added B_2_O_3_ will absorb excess free oxygen to some extent, entering the network structure in [BO_4_], thereby increasing the density of the glass network structure and enhancing its strength.

A2 to A4: Combining XRD data, we found that the crystallization amount of quartz increases with the variation of composition points, which leads to a decrease in the strength of the glass. Meanwhile, the content of silicon dioxide involved in the glass network structure decreases. The [SiO_4_] tetrahedron is a fundamental component of glass formation, which interconnects through shared oxygen atoms in the glass network, forming a continuous three-dimensional random network. The decreased involvement of [SiO_4_] tetrahedral units in network construction implies that the random network becomes sparser, consequently reducing the mechanical strength of the glass particles themselves. Thus, the optimal single-particle compressive strength of abrasive grains shows a decreasing trend. On the other hand, according to the boron anomaly phenomenon, the decrease in sodium oxide content reduces the number of non-bridging oxygens in the network structure, coupled with a further increase in boron oxide content, which to a certain extent also increases the number of [BO_3_] triangle, while decreasing the number of [BO_4_] tetrahedra. This results in an increase in [BO_3_] triangles, namely layered structures in the network structure, thus reducing the uniformity of the glass phase and weakening the mechanical strength of the structure.

**Figure 7:**
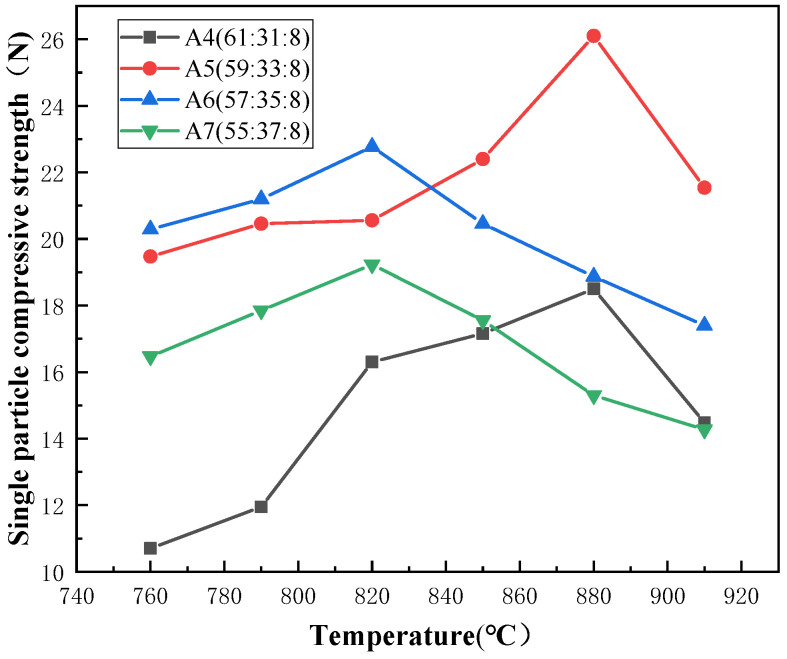
Single particle compressive strength of stacked abrasives prepared at A4 to A7.

**Figure 8:**
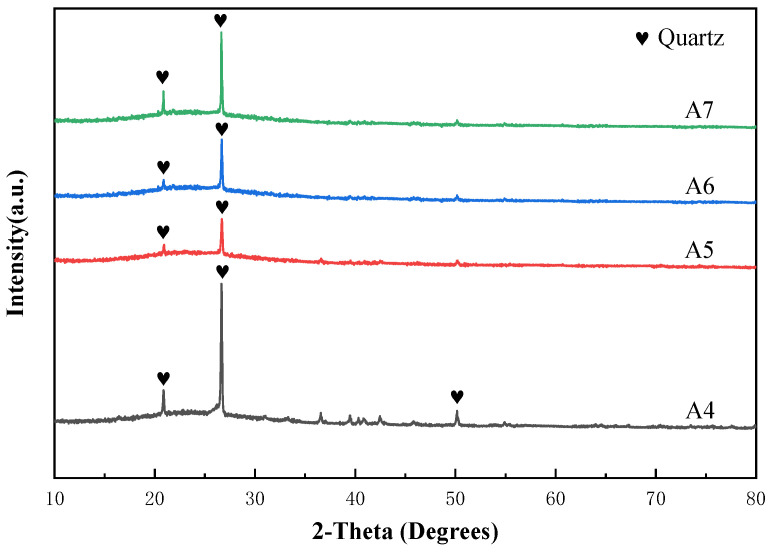
XRD patterns of stacked abrasives prepared at A4 to A7 sintered at the 850 °C.

From Figure 7, it can be observed that under the constant sodium oxide content, the decrease in silicon dioxide content and the increase in boron oxide content result in an initial increase, followed by a decrease in the optimal single-particle compressive strength. It reaches a maximum value of 26.10 N at point A5, while the optimal sintering temperature shows a decreasing trend. From Figure 8, the precipitation of quartz crystals undergoes a significant decrease at point A5 followed by a subsequent rising trend with the variation of composition points.

A4 to A5: The optimal single-particle compressive strength at A5, compared to the composition point at A4 (18.50 N), has significantly increased. At this component point A4, the ratio of n (Na_2_O/B_2_O_3_) has reached 8:31, indicating that the content of B_2_O_3_ has greatly exceeded that of Na_2_O. According to the theory of glassy amorphous network structure, at A4, the free oxygen provided by sodium oxide in the glass structure is no longer sufficient to allow the newly added B_2_O_3_ to enter the glass structure in tetrahedral form [BO_4_]. Therefore, the newly added B_2_O_3_ will form a layered single-component glass structure in the form of the boron-oxygen triangle [BO_3_], reducing the overall homogeneity of the glass phase. Additionally, due to the molecular forces (van der Waals forces) between the layers of planar B_2_O_3_ structures, the increase in B_2_O_3_ content will lead to a decrease in the mechanical strength of the glass. Although previous analysis indicates that the increase in B_2_O_3_ content will unilaterally decrease the mechanical strength of the glass, the strength of the vitrified bond is not only influenced by the differences in the glass network structure but also affected by the crystallization. In cases where the amount of crystallization is severe, crystallization becomes the primary factor limiting the compressive strength of the stacked abrasives. From Figure 8, it can be observed that the quartz diffraction peak of A4 is already very intense, indicating that the crystallization amount of quartz in A4 is considerable. This significantly reduces the single particle compressive strength of the stacked abrasives at A4. In the transition from A4 to A5, we observed a significant decrease in the amount of quartz crystallization compared to A4. This greatly reduces the formation of microdefects caused by crystals in the glass structure, thereby enhancing the uniformity of the glass structure. Therefore, although the increase in the planar structure of [BO_3_] in A5 will change the glassy amorphous network structure of the vitrified bond and have a negative impact on the compressive strength of the abrasive, the significant reduction in the crystallization amount, which is the primary factor limiting the compressive strength, still results in an increase in the maximum compressive strength of the stacked abrasives at A5.

A5 to A7: During the variation process from A5 to A7 composition points, although the precipitation of silicon dioxide has significantly decreased compared to the A4 composition point, the gradually rising trend leads to a decrease in the uniformity of the glass structure, resulting in a decrease in strength. Furthermore, with the increase in boron oxide content, while the silicon dioxide content decreases, more [BO_3_] triangle enters the glass network structure, leading to reduced uniformity and increased fluidity of the glass phase. Therefore, the optimal sintering temperature and the optimal single particle compressive strength show a downward trend.

#### 3.2.2. Microstructure of Stacked Abrasives at Different Sintering Temperatures

**Figure 9:**
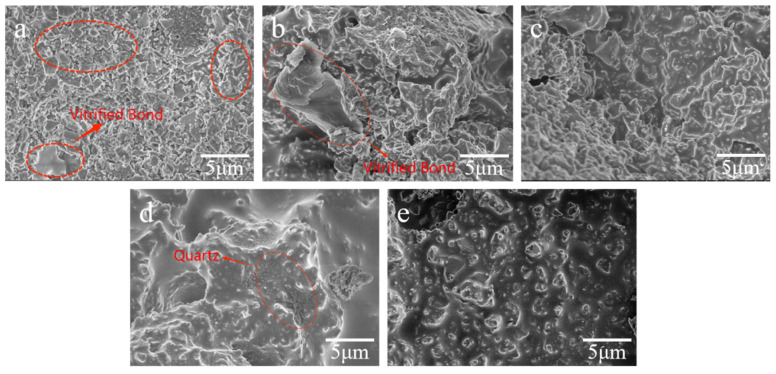
SEM of stacked abrasives prepared with A2 component point sintered at different temperatures: (**a**) 760 °C, (**b**) 790 °C, (**c**) 820 °C, (**d**) 850 °C, and (**e**) 880 °C.

It can be observed from Figure 9a,b that the uneven distribution of the binder between the abrasive grains indicates poor fluidity of the binder, which fails to adequately encapsulate the abrasive grains. In Figure 9c with the temperature increasing to 820 °C, it is observed that although there is a small amount of silicon dioxide crystals generated, the fluidity of the bond is high enough to allow the bond melt to completely encapsulate the abrasive grains. The high temperature melt of the bond flows better between the alumina abrasive grains, enveloping them comprehensively, thus achieving a uniform and dense structure of the stacked abrasive grains. In addition, the phase analysis shows that the cristobalite crystals have disappeared at this temperature, and the amount of quartz crystallization is small, which has a weak impact on the compressive strength. As the sintering temperature reaches 850 °C, the fluidity of the sol-gel bond further increases, while silicon dioxide small crystals can already be clearly observed precipitating on the surface of the binder from Figure 9d. This affects the uniformity of the glass network structure, resulting in a decrease in the strength of the stacked abrasive grains. When the temperature continues to rise to 880 °C, on one hand residual active components in the bond undergo decomposition and some [BO_3_] begin to volatilize, resulting in foaming as evidenced by the presence of some pores between the alumina abrasive grains from Figure 9e. On the other hand, excessive fluidity of the bond leads to its aggregation under the influence of gravity, preventing uniform dispersion among the abrasive grains, thus causing a decrease in the strength of the stacked abrasive grains.

#### 3.2.3. Stacked Abrasives under Different Additions of Vitrified Bonds

**Figure 10:**
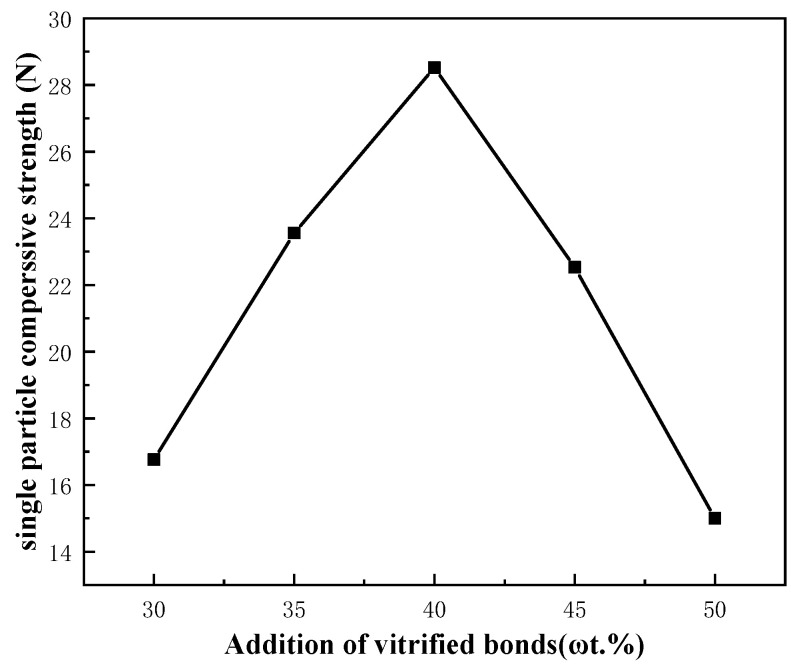
Single particle compressive strength of stacked abrasives with different additions of vitrified bonds.

Figure 10 demonstrates that the compressive strength of a single particle was only 16.76 N at 30% of the bond content and increased to a maximum value of 28.56 N at 40%. Subsequently, as the bond content continues to increase to 45% and 50%, the compressive strength of the abrasives gradually decreases to 22.53 N and 15.00 N, respectively, and the optimum amount of bond should be added at a level of 40%.

**Figure 11:**
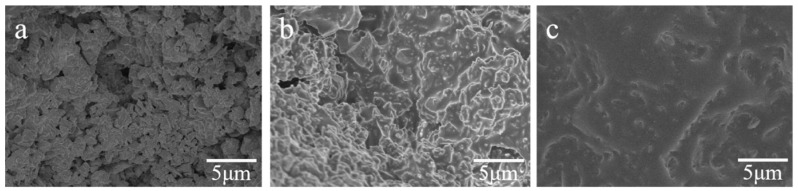
SEM of stacked abrasives with different additions of vitrified bonds: (**a**) (30%), (**b**) (40%), and (**c**) (50%).

In order to explain why the stacked abrasives, show the highest single particle compressive strength at 40% bond addition, it is necessary to analyze the SEM of stacked abrasives with different additions of vitrified bonds. Figure 11 shows that when the binder content is 30% (a), although the lower amount of binder leads to less precipitation of quartz crystals, thereby increasing the mechanical strength of the vitrified bond, it can be observed from figure (a) that there is a lack of bonding between some alumina abrasives, resulting in numerous voids and cracks. These defects act as stress concentration points, promoting crack propagation, indicating that a bond content of 30% is far from sufficient to completely coat the surface of the alumina micro-powder particles, resulting in low compressive strength of the stacked abrasives. When the bond content is 40% (b), the vitrified bond completely coats the abrasives, and the outlines of the abrasives are still clearly visible. This indicates that the bond content is appropriate, and therefore, at this point, the stacked abrasives achieve the optimal single particle compressive strength. When the bond content is 50% (c), the increased participation of the bond in sintering in the stacked abrasives will inevitably lead to more precipitation of the quartz crystals, resulting in a decrease in the mechanical strength of the vitrified bond. On the other hand, it can be seen from the figure that although a large amount of binder completely coats the abrasives, it almost submerges the alumina abrasives. Excessive vitrified bond leads to an imbalance in the ratio of bond to abrasives in the abrasive, causing the structure of the alumina abrasive grains to become loose, as excess binder fills the gaps in the abrasive, reducing the density between abrasives.

### 3.3. Stacked Abrasives Prepared by Dry Mixing Method

**Figure 12:**
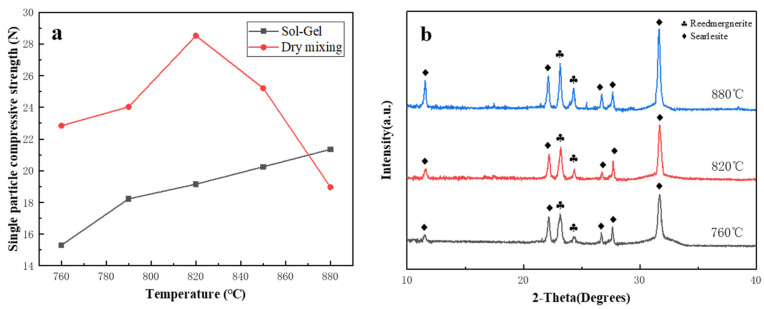
Single particle compressive strength of stacked abrasives prepared by the sol-gel and dry mixing methods (**a**) and XRD patterns of stacked abrasives prepared by dry-mixing method (**b**).

Vitrified bonds and alumina stacked abrasives were prepared using traditional dry mixing and sol-gel methods, respectively, according to the A2 composition ratio. As shown in Figure 12, within the temperature range of 760 °C to 880 °C, the vitrified bonds prepared by the traditional dry mixing method still remain in the stage of generating reedmergnerite and searlesite. Moreover, within the low-temperature sintering range, the compressive strength of the sol-gel bonds is significantly higher than that of the ceramic binders prepared by the traditional dry mixing method. This is because glass bonds, due to their amorphous structure, can often achieve faster bonding with abrasive grains during the sintering. Vitrified bonds, prepared by the sol-gel method at low temperatures, exhibit higher fluidity, enabling better filling of gaps between abrasive grains during sintering, thereby improving the density and uniformity of stacked abrasives. However, vitrified bonds prepared by the traditional dry mixing method typically have higher melting points due to their crystalline structure. To melt them and complete sintering, it is necessary to increase the sintering temperature. Additionally, during the high-temperature sintering of crystalline binders, time is required for the growth and arrangement of crystal structures to ensure strong bonding between materials. This process is relatively slow and requires a longer time to complete, implying higher energy consumption and longer sintering time.

### 3.4. Compressive Strength and Microstructure of Diamond Stacked Abrasives

**Figure 13:**
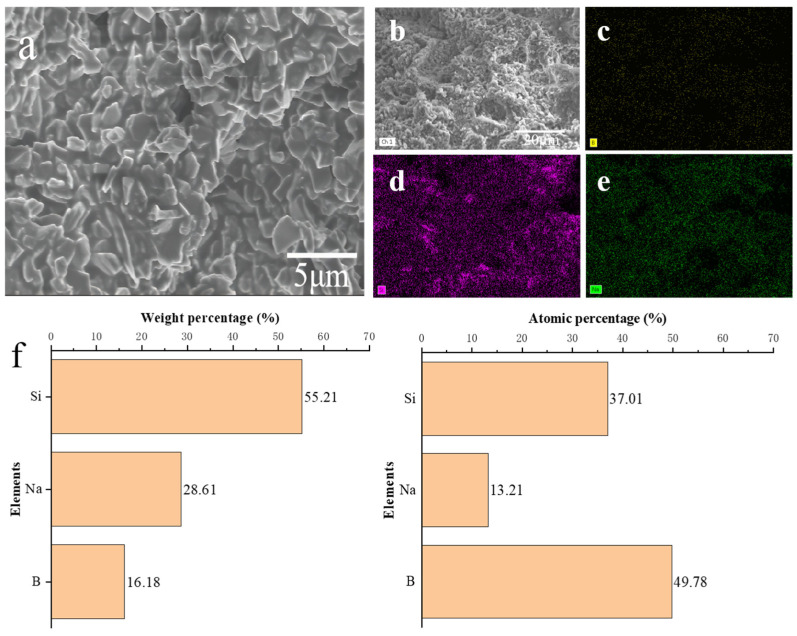
SEM (**a**,**b**) and corresponding EDS of diamond stacked abrasives: (**c**) B, (**d**) Si, (**e**) Na, and (**f**) elemental composition.

Diamond stacked abrasives were prepared by the optimum A2 composition point of sol-gel vitrified bond with diamond micropowder and sintered at 820 °C. After the compressive strength test, the single particle compressive strength of the diamond stacked abrasives reached 28.14 N, which was slightly lower than that of corundum stacked abrasives. Zhang et al. [32] also utilized the molding and crushing method to prepare CBN stacked abrasives, but unlike this study, they employed the melt quenching method to prepare the vitrified bond. In comparison, the sintering temperature of the stacked abrasives in our study decreased by approximately 100 °C, while the compressive strength increased by about 10 N. This also indicates the advantages of using the sol-gel method to prepare low-temperature vitrified bond. Cao et al. [33] also prepared the vitrified bond and stacked abrasives using melt quenching and molding and crushing methods, respectively, and in their study the compressive strength of the corundum stacked abrasives could be maintained at 26 N at a sintering temperature of 760 °C. Although the sintering temperature of the stacked abrasives decreased by approximately 60 °C compared to our study, the vitrified bond used in their research is a six-component oxide system. Therefore, the composition point is relatively mature, and dispersion strengthening, and microcrystalline toughening are utilized to enhance the compressive strength of the abrasives. However, the bond in our study is only a ternary system. Therefore, there is reason to believe that adding some intermediate oxide on the basis of the A2 ternary composition point can further reduce the sintering temperature and increase the single particle compressive strength. At the same time, it can be seen from Figure 13 that the bond has good wrapping performance for diamond micro-powder, and there are few pores connected to the inside of the stacked abrasives. Meanwhile, energy spectrum scanning was performed on the surface morphology of the diamond stacked abrasives. The EDS show that the elements B, Si, and Na are evenly distributed on the surface of the stacked abrasives, thereby confirming that the sol-gel vitrified bond at the A2 composition point can achieve good wetting and encapsulation on the surface of diamonds.

## 4. Conclusions

This study utilized the sol-gel method to prepare Na_2_O-B_2_O_3_-SiO_2_-based vitrified bond powders with different composition ratios and employed the molding and crushing method to prepare stacked abrasives of corundum and diamond. Innovatively, the study analyzed the effects of different compositional points of sol-gel vitrified bonds, as well as sintering temperature and bond addition amount, on the single particle compressive strength and the microstructure of stacked abrasives.

1. The heat treatment temperature of the ternary system vitrified bond powder prepared by the sol-gel method should be set at 600 °C to ensure complete decomposition of NaNO_3_.
2. When the composition ratio of the ceramic bond is A2: n(SiO_2_):n(B_2_O_3_):n(Na_2_O) = 65:23:12, the sintering temperature is 820 °C, and the amount of bond added is 40%, the prepared corundum stacked abrasives can reach the maximum single grain compressive strength which is 28.56 N, and the diamond stacked abrasives can reach the single grain compressive strength of 28.14 N.
3. Vitrified bonds were prepared using the dry mixing method at the same composition point A2. However, within the temperature range of 760 °C to 880 °C, it was found to be merely a process of the crystallization of sodium borosilicate, and the strength of the agglomerated abrasives sintered at low temperatures was far inferior to that of sol-gel vitrified bonds stacked abrasives.
4. This paper selects the most basic ternary oxide system as the vitrified bond. Therefore, it is possible to continue using the sol-gel method to add some intermediate oxides such as ZnO and Al_2_O_3_ to the vitrified bond with A2. This addition aims to further reduce the sintering temperature of the stacked abrasives while increasing the compressive strength. Furthermore, the effect of the addition ratio of intermediate oxides on the thermal expansion coefficient of the ceramic binder can be discussed in order to achieve the most suitable match with the abrasive micro-powder, thereby preparing stacked abrasives with excellent performance for application.

## Figures and Tables

**Table 1:** Chemical compositions of the vitrified bonds (molar ratio).

Sample Composition Points	SiO_2_, %	B_2_O_3_, %	Na_2_O, %
A1	67	19	14
A2	65	23	12
A3	63	27	10
A4	61	31	8
A5	59	33	8
A6	57	35	8
A7	55	37	8

A1 to A4: the oxide ratios vary regularly. A4 to A7: the Na_2_O content remains unchanged, the B_2_O_3_ content increases and the SiO_2_ content decreases.

## Data Availability

Data are contained in the article.
